# Facile and Efficient Fabrication of Bandgap Tunable Carbon Quantum Dots Derived From Anthracite and Their Photoluminescence Properties

**DOI:** 10.3389/fchem.2020.00123

**Published:** 2020-02-28

**Authors:** Jianbo Jia, Yue Sun, Yaojie Zhang, Quanrun Liu, Jianliang Cao, Guangxu Huang, Baolin Xing, Chuanxiang Zhang, Lina Zhang, Yijun Cao

**Affiliations:** ^1^Henan Key Laboratory of Coal Green Conversion, College of Chemistry and Chemical Engineering, Henan Polytechnic University, Jiaozuo, China; ^2^Henan Province Industrial Technology Research Institute of Resources and Materials, Zhengzhou University, Zhengzhou, China

**Keywords:** carbon quantum dots, coal, photoluminescent, anthracite, bandgap engineering

## Abstract

Low-cost and earth-abundant coal has been considered to have a unique structural superiority as carbon sources of carbon quantum dots (CQDs). However, it is still difficult to obtain CQDs from raw coal due to its compactibility and lower reactivity, and the majority of the current coal-based CQDs usually emit green or blue fluorescence. Herein, a facile two-step oxidation approach (K_2_FeO_4_ pre-oxidation and H_2_O_2_ oxidation) was proposed to fabricate bandgap tunable CQDs from anthracite. The K_2_FeO_4_ pre-oxidation can not only weaken the non-bonding forces among coal molecules which cause the expansion of coal particles, but also form a large number of active sites on the surface of coal particles. The above effects make the bandgap tunable CQDs (blue, green, or yellow fluorescence) can be quickly obtained from anthracite within 1 h in the following H_2_O_2_ oxidation by simply adjusting the concentration of H_2_O_2_. All the as-prepared CQDs contain more than 30 at% oxygen, and the average diameters of which are <10 nm. The results also indicate that the high oxygen content only can create new energy states inside the band gap of CQDs with average diameter more than 3.2 ± 0.9 nm, which make the as-prepared CQDs emit green or yellow fluorescence.

## Introduction

Carbon quantum dots (CQDs), new zero-dimensional carbon nanomaterials whose size are similar with conventional semiconductor quantum dots but the skeleton is based on carbon, have attracted tremendous research interest after been found (Jaleel and Pramod, 2018; Kaur et al., 2018; Riyanto et al., 2019; Wang et al., 2019; Zhou et al., 2019). And CQDs have been expected to have large potential application in biomedicine (Jaleel and Pramod, 2018), photovoltaic device (Li X. et al., 2015; Kaur et al., 2018), ion detection (Wu et al., 2014; Arumugam and Kim, 2018; Li et al., 2018; Wang et al., 2018; Zhang et al., 2018; Omer et al., 2019), photocatalysis (Yu et al., 2014; Azimirad et al., 2017; Zhang B. et al., 2017; Syed et al., 2019), and other fields due to their fascination optical and electro-optical properties (Shao et al., 2016; Pramanik et al., 2018).

Coal is consist of angstrom-sized or nanometer-sized crystalline carbon linked by amorphous carbon and polymerized aromatic hydrocarbon (Thiyagarajan et al., 2016). These crystalline carbon domains are abundant in coal and the size of them meet requirements of CQDs (Dong et al., 2014; Hoang et al., 2018). Additionally, the cheapest price and substantial deposits of coal, in contrast to crystalline carbon such as graphene, carbon tubes and fullerenes, have attracted tremendous interest and efforts in developing preparation methods of CQDs from coal. Up to now, CQDs have been successfully prepared from coal by different methods (Ye et al., 2013; Dong et al., 2014; Hu et al., 2015, 2016; Li M. et al., 2015; Sasikala et al., 2016; Li et al., 2017; Liu X. et al., 2018; Saikia et al., 2019). Ye et al. (2013) employed concentrated sulfuric acid and nitric acid to exfoliate CQDs from coal at 100° or 120°C for 24 h. Similarly, CQDs were obtained through refluxing coal in 5 M HNO_3_ at 120°C for 12 h (Dong et al., 2014). However, there are some drawbacks to the above methods, such as the longer reaction time and the inherent difficulty in separation of CQDs from the mixture which contains a large amount of inorganic salts that formed during the neutralization phase via the addition of bases. Hence, in order to optimizing the preparation conditions of CQD from coal, selective depolymerization of coal in an oxidizing supercritical fluid was proposed by Sasikala et al. (2016). They isolated CQDs in supercritical water under the conditions of 400°C and 25 MPa within 2 h. Although this way could observably shorten the time to prepare CQDs, the unattainable reaction conditions hampered the large scale preparation of CQDs. Whereupon, greener oxidants (H_2_O_2_, O_3_) were utilized to produce CQDs from coal under milder reaction conditions (Hu et al., 2016; Liu X. et al., 2018).

Despite these efforts, most current CQDs prepared from coal show green or blue fluorescent. And the difficulties in obtaining a defined and desired bandgap have largely hindered the applications of CQDs for a particular purpose (Yan et al., 2018). Hence, in order to obtain narrow bandgap CQDs (yellow to red fluorescent) from coal, there have been more efforts to tailor bandgap of CQDs. The bandgap of CQDs opening is due to the quantum confinement effect (Pan et al., 2010). Consequently, tuning the lateral size of CQD is one of the common strategies for narrowing bandgap of CQD. Different nanometer-sized CQDs were prepared from various coal or coke which possess different-sized graphene domains by strong acidic oxidation (Ye et al., 2013; Hu et al., 2015). In addition, different nanometer-sized CQDs can also be prepared solely from anthracite in concentrated H_2_SO_4_ and HNO_3_, and the size control of CQDs was achieved through cross-flow ultrafiltration, controlling the reaction temperature of the oxidation process or conjugating pristine CQDs with poly aromatic rings (Ye et al., 2015; Yan et al., 2018). Another approach for narrowing the bandgap is forming intramolecular Z-schemes structure via functionalization of pristine CQDs with electron-donating chemical groups (Yan et al., 2018). Much progress has been made, but many more problems need solving before CQDs with varying bandgaps can be feasibly produced from coal in large-scale, such as long production phases, critical synthesis conditions or expensive reagents. Therefore, a simple, fast and facile synthesis method is still highly desirable.

Here we report a two-step, facile and fast method to fabricate CQDs with varying bandgaps solely from anthracite. In the first step, which is called pre-oxidation stage, anthracite was oxidized with potassium ferrate K_2_Fe^VI^O_4_ as an oxidant in H_2_SO_4_ medium to obtain the oxidized coal. The sp^3^-hybridized carbon atoms can be selectively oxidized and abundant oxygen-containing groups were produced after K_2_FeO_4_/H_2_SO_4_ treatment (Zhang and Xu, 2015). This can substantially improve the chemical reaction activity and wet ability of anthracite. In the second step, CQDs with varying bandgaps can be fast fabricated from the oxidative coal using H_2_O_2_ as an oxidant by simply controlling the concentration of H_2_O_2_.

## Experimental

### Materials

Anthracite collected from Taixi (Inner Mongolia Province, China) without pretreatment was crushed and ground to powder (about 200 mesh). H_2_O_2_ (30 wt.%) and H_2_SO_4_ (98%) were purchased from Sinopharm Chemical Reagent Co., Ltd., China. K_2_FeO_4_ was purchased from Shanghai Mecoxlane International MailorderCo., Ltd., China. Polyethersulfone filter membranes(0.22 μm) were purchased from Jinteng Experimental Equipment Co., Ltd., China. All reagents were used as received unless otherwise noted. Deionized water was used for all experiments.

### Pre-oxidation of Anthracite

Two gram anthracite and 100 ml of concentrated H_2_SO_4_ were mixed in a 250 ml flask. Then 2.5 g potassium ferrate was slowly added in small doses to avoid overheating. The reaction mixture was kept at 40°C for 1 h under magnetic stirring. Once the reaction had finished, the mixture was centrifuged to recycle the concentrated H_2_SO_4_. The precipitate was poured in to 100 ml water and stand for 30 min. The oxidized coal was obtained by repeated centrifugation and washing with water until the pH of the supernatant approached 7.

### Synthesis of CQDs

One gram oxidized coal was mixed with 50 ml H_2_O_2_ solution, then the obtained mixture was stirred and reacted at 100°C for 1 h. Subsequently, the unreacted coal was removed via centrifugation at 8,000 rpm for 5 min. The supernatant was then filtered through a 0.22 μm filter membrane to remove the larger fragments and the filtrate was dialyzed in 1,000 Da dialysis bag. After purification, the filtrate was freeze-dried to obtain solid CQDs. In order to adjust the bandgap of CQDs, only the concentration of H_2_O_2_ was changed from 30 to 10%, and the other experimental conditions remain unchanged.

### Characterization

Transmission electron microscopy (TEM) images were conducted with a Tecnai G2 F20 instrument (FEI, USA) operated at 200 kV. The scanning electron microscope (SEM) was performed on a Quanta FEG 250 field-emission SEM system (FEI, USA). The fluorescence spectra were measured by a Cary Eclipse spectrophotometer (Varian, USA). X-ray photoelectron spectroscopy (XPS) measurement was performed on an ESCALAB250 Xi photoelectron spectrometer (Thermo Fisher Scientific, USA). The Fourier-transform infrared spectroscopy (FT-IR) and Ultraviolet-visible (UV–Vis) absorption spectroscopy were obtained from a VERTEX 70 FTIR spectrometer (Bruker, Germany) and a Pgeneral TU-1810 spectrometer (Pgeneral, China), respectively. Raman spectra were recorded using a microscopic confocal raman spectrometer (Renishaw, UK) with an argon ion laser (λ = 514 nm) at ambient temperature. The X-ray diffraction (XRD) patterns of all samples were recorded on a Bruker D8 Advance (Bruker, Germany) with a Cu Kα X-ray radiation source (λ = 0.15418 nm), and the scattering angles (2θ) range from 10° to 80°.

## Results and Discussion

### Characterization of Oxidized Coal and Preparation Principle Analysis of Bandgap Tunable CQDs

**Figure 1:**
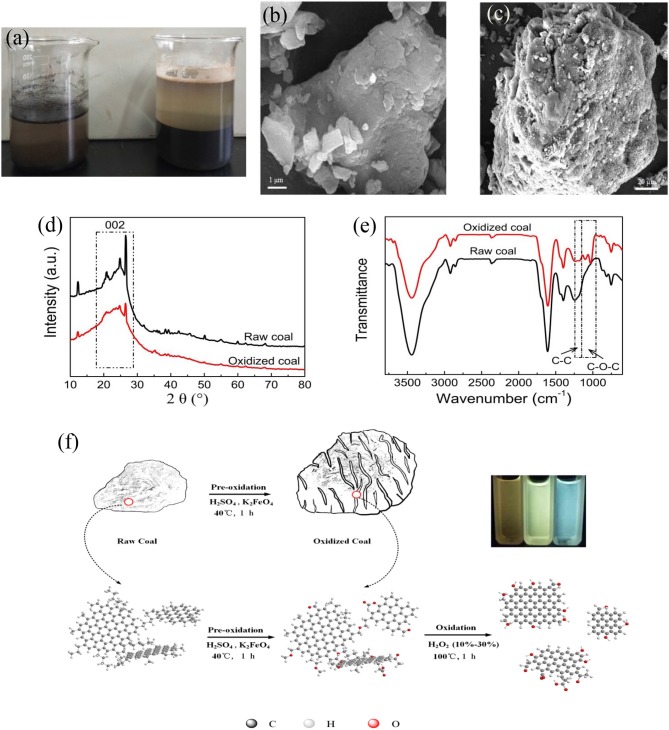
**(a)** The disperse of anthracite in water (left: raw coal, right: oxidized coal). SEM images of **(b)** raw coal and **(c)** oxidized coal. **(d)** XRD and **(e)** FTIR spectra of raw coal and oxidized coal. **(f)** Schematic illustration of CQDs synthesis.

Raw anthracite was oxidized with K_2_FeO_4_/H_2_SO_4_ to improve its surface reactivity. As shown in Figure 1a, the wet ability of pulverized coal is very poor, and most of which floated on the surface of water before oxidized. However, pulverized coal could be very well-moistened with water and the volume of raw coal expanded significantly after the pre-oxidation, indicating that the pre-oxidation has the powerful influence on the structure and properties of anthracite. SEM was used to observe the microscopic morphology change of anthracite before and after the pre-oxidation (Figures 1b,c). Compared with the compact structure of raw coal, there are lots of crevices or pores on the surface of the oxidized coal, which may be offer much more active sites to react with H_2_O_2_ reactant used subsequently for fabrication of CQDs. To obtain more information about the structure change of anthracite after the pre-oxidation, original and oxidized samples were analyzed by the X-ray diffraction (XRD) and Fourier transform infrared spectroscopy (FTIR). The XRD spectra (Figure 1d) show that one broad peak observed at 24.5° is attributed to the 002 plane of graphite lattice for both original and oxidized samples (Duraia et al., 2018; Liu Y. et al., 2018). The position and full width at half maximum intensity of 002 peak have little change before and after the pre-oxidation, indicating that the crystalline carbon domains of anthracite were not corrupted in the process of pre-oxidation. The FTIR spectra are shown in Figure 1e. Major structural changes during pre-oxidation for anthracite mainly occurred around 1,000 and 1,300 cm^−1^ region. The intensity of 1,150–1,250 cm^−1^ assigned to saturated aliphatic skeletal C-C vibration was found to be weaker and there was a group of peaks in the region 1,150–950 cm^−1^ due to stretching vibration of C-O (C-O-C or phenolic) for oxidized coal (Okolo et al., 2015; Xing et al., 2017; Qiu et al., 2020), suggesting that a large number of oxygen-containing functional groups were formed and a part of aliphatic carbon atoms were consumed during the pre-oxidation process.

The above results can be interpreted as the selective oxidation of K_2_FeO_4_/H_2_SO_4_, namely the sp^3^ C-C bonds were oxidized while the C = C bonds remained intact (Zhang and Xu, 2015). H_2_SO_4_ and K_2_FeO_4_ can quickly intercalate into the coal molecule interval. The aliphatic portions were oxidized while the oxidant reacts with H^+^ or water to produce a lot of oxygen gas (Peng et al., 2015), these results can break the non-covalent interactions (hydrogen bonds, π-π interactions, van der Walls interactions and electrostatic interactions) (Niekerk et al., 2010) and cause the significant swelling bulk of powdered coal. Overall, compared to original coal, the oxidized coal possesses higher reactivity in reaction with H_2_O_2_. The entire fabrication process of CQDs requires only 1 h, and the as-prepared CQDs with varying bandgaps were obtained by solely controlling the concentration of H_2_O_2_. The CQDs formation mechanism is shown in Figure 1f.

### Characterization of Bandgap Tunable CQDs

**Figure 2:**
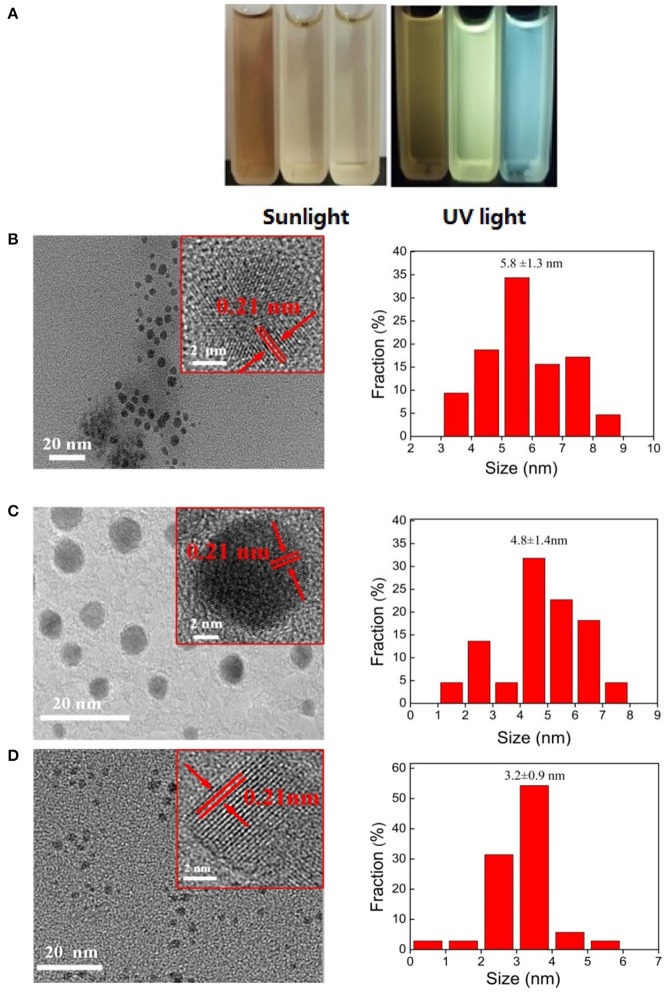
**(A)** Aqoeous solution of CQDs under sunlight and UV light (the left cuvette is CQDs-N10-Y solution, the middle cuvette is CQDs-N20-G solution and the right cuvette is CQDs-N20-G solution). The TEM images, HRTEM images, and size distributions of **(B)** CQDs-N10-Y, **(C)** CQDs-N20-G, and **(D)** CQDs-N30-B.

The CQDs synthesized with different concentration of H_2_O_2_ at 100°C are denote as CQDs-Nx-y where “N” signifies “Concentration,” “x” signifies the concentration of H_2_O_2_ and “y” signifies the fluorescent color of CQDs solution under UV lamp (365 nm) irradiation, such as yellow, green, and blue are represented by Y, G and B, respectively. The change of CQDs in bandgap is visualized in Figure 2A, where the as-prepared CQDs solutions emit from blue to yellow under UV lamp (365 nm) irradiation. An of the interesting finding was that the bandgap of CQDs is red-shifted from blue to yellow with decreasing of H_2_O_2_ concentration from 30 to 10%. The production yields of CQDs-N10-Y, CQDs-N20-G, and CQDs-N30-B are 18.9, 12.6, and 4.3%, respectively. The production yield of CQDs is the ratio between the obtained CQDs solid powder and the amount of coal. The microstructure of the as-prepared CQDs was investigated by transmission electron microscopy (TEM). The TEM images and the size distributions of the as-prepared CQDs are shown in Figures 2B–D and the high-resolution TEM (HRTEM) of the as-prepared CQDs is inset in the corresponding TEM. It was found that the diameters of the as-prepared CQDs were relatively uniform and all the as-prepared CQDs have a quasi-spherical shape. The HRTEM images reveal that the as-prepared CQDs have highly crystalline structure with a lattice spacing of ca. 0.21 nm, which is corresponding to the (100) diffraction facets of graphite carbon (Tian et al., 2017). The size distributions of CQDs-N10-Y, CQDs-N20-G, and CQDs-N30-B are 5.8 ± 1.3, 4.8 ± 1.4, and 3.2 ± 0.9 nm, respectively.

**Figure 3:**
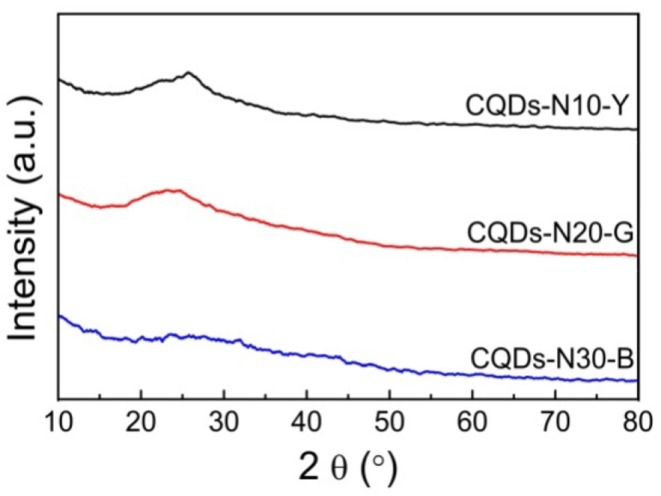
XRD patterns of CQDs-N10-Y, CQDs-N20-G, and CQDs-N30-B.

The XRD patterns of the as-prepared CQDs (Figure 3) show that one broad peak (about 25°) ascribed to the 002 plane of graphite lattice is observed, and this peak of CQDs-N30-B is almost unseeable due to its smaller size. The CQDs could also be prepared by directly oxidation of anthracite with H_2_O_2_, but the diameters of which are mainly distributed from 1 to 3 nm significantly smaller than that of the CQDs prepared in this work and they emit blue under UV light (Hu et al., 2016). This indicates the pre-oxidation of anthracite plays a key role in the bandgap adjustment of CQDs which can shorten the oxidative time to avoid the excessive oxidation of crystalline carbon domains of anthracite. In general, as the size of CQDs increases, the bandgap narrows. Therefore, at least initially, this work was designed to tune the bandgap by changing the size of CQDs, however the average diameters of the as-prepared CQDs in this work are all <10 nm. In the literature (Pan et al., 2010), the CQDs with 9.6 nm average diameter were fabricated from graphene oxide by hydrothermal treatment at 200°C, and they emit bright blue under UV light, indicating that the major factor adjusting the bandgap of the as-prepared CQDs isn't the size.

**Figure 4:**
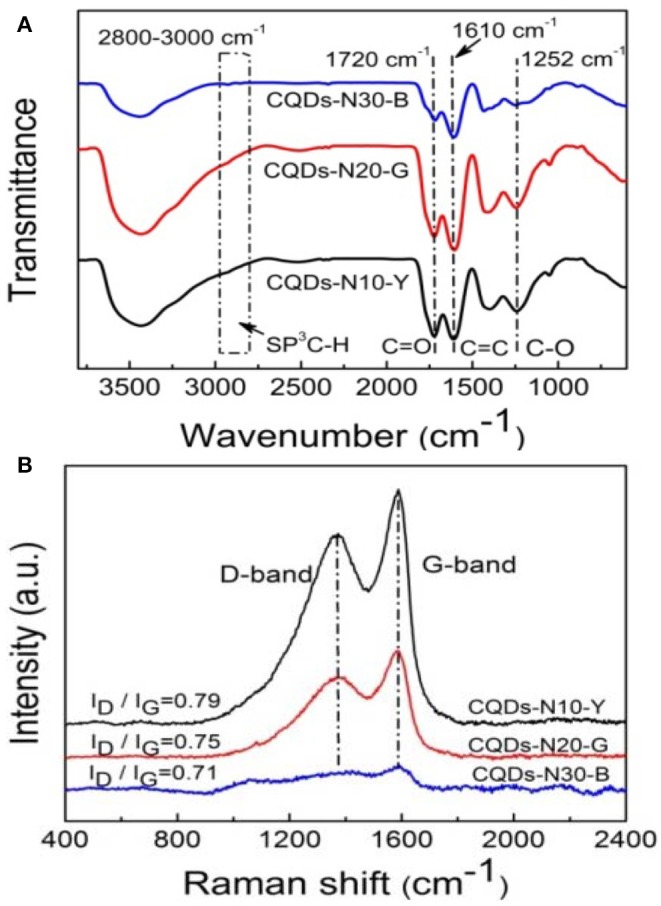
**(A)** FTIR and **(B)** Raman spectra of CQDs-N10-Y, CQDs-N20-G, and CQDs-N30-B.

To further understand the effect of reaction conditions on the functional groups, FTIR, Raman and X-ray photoelectron spectroscopy (XPS) and were applied to investigate the structure of CQDs. The FTIR spectra of CQDs are shown in Figure 4A. The absorption peaks at 2,800–3,000 cm^−1^ are assigned to stretching vibrations of aliphatic C-H. The strong absorption peak at 1,610 cm^−1^ are in associated with stretching vibration of aromatic C=C. Compared to the oxidized coal, absorption peaks (2,800–3,000 cm^−1^) almost disappear in the FTIR spectra of CQDs, indicating the aliphatic carbon chains which link the crystalline carbon domains of coal have be selectively oxidized into CO_2_ and H_2_O by H_2_O_2_ (Hu et al., 2016). Besides, the peaks at 1,720 cm^−1^ and 1,252 cm^−1^ are attributed to the C=O stretching, the O-H deformation and C-O stretching of carboxylic acid, respectively, which make CQDs hydrophilic and stable dispersion in water. The Raman spectra of the as-prepared CQDs (Figure 4B) reveal a D band at 1,367 cm^−1^ and a G band at 1,587 cm^−1^, which correspond to disordered structures and graphitic structures, respectively, of carbon materials (Ding et al., 2015; Huang et al., 2020). The intensity ratio for the D to G bands can be used to reflect the level of disorder in carbon materials (Ma et al., 2019). The values of I_D_/I_G_ for CQDs-N10-Y, CQDs-N20-G, and CQDs-N30-B are 0.79, 0.75, and 0.71, respectively, suggesting the as-prepared CDQs possess much more ordered graphite structures with decreasing of size of CQDs.

**Figure 5:**
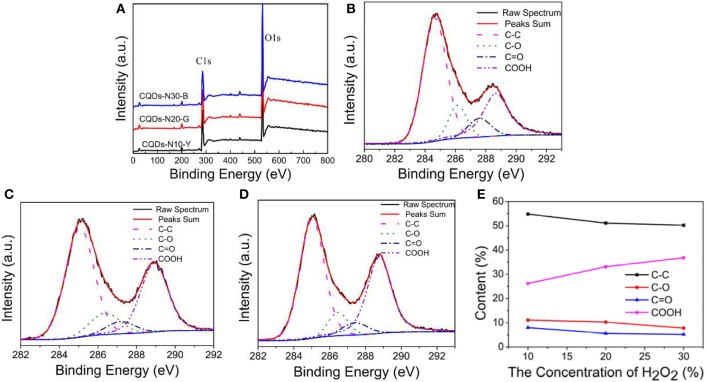
**(A)** XPS survey spectra of CQDs-N10-Y, CQDs-N20-G, and CQDs-N30-B. C 1s high resolution XPS spectra of **(B)** CQDs-N10-Y, **(C)** CQDs-N20-G, and **(D)** CQDs-N30-B. **(E)** Summary of relative percentage of different functional groups on the surface of CQDs from **(B)** to **(D)**.

The chemical structures at the surface of the as-prepared CQDs were further investigated by X-ray photoelectron spectroscopy (XPS) analyses. The XPS survey spectra of CQDs (Figure 5A) show that the as-prepared CQDs primarily consist of carbon and oxygen (Zhang Q. et al., 2017, 2019; Zeng et al., 2020). It can be seen that the oxygen content gradually increases from 34.4 to 42.6% with the increasing of the H_2_O_2_ concentration in Table 1, indicating the higher the concentration of H_2_O_2_, the stronger the oxidation ability, due to the increase of the number of·OH radical. As shown in Figures 5B–D, the high resolution C 1s spectra of CQDs can be conceived into four peaks at 285 eV (C-C/C = C/C-H), 286.3 eV (C-O), 287.4 eV (C = O), and 288.8 eV (COOH) (Roy et al., 2014; Shi et al., 2015; Moonrinta et al., 2018; Pacquiao et al., 2018), suggesting the presence of large quantities of oxygen-containing functional groups, especially carboxyl groups. The relative abundances of these components are summarized in Figure 5E.

**Table 1:** Element content of the as-prepared CQDs calculated based on XPS analysis (atom %).

**Samples**	**C**	**O**
CQDs-N10-Y	63.8	34.4
CQDs-N20-G	57.1	40.9
CQDs-N30-B	55.9	42.6

### Optical Properties of Bandgap Tunable CQDs

**Figure 6:**
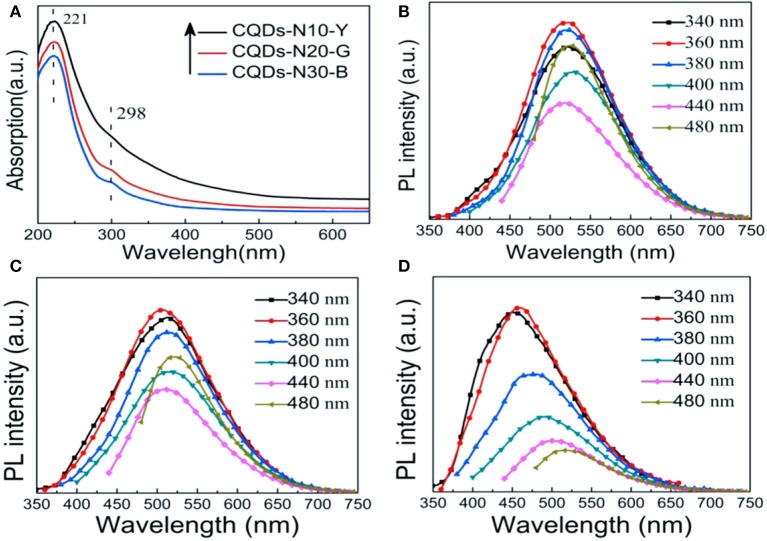
**(A)** UV-vis absorption spectra of CQDs-N10-Y, CQDs-N20-G, and CQDs-N30-B. Photoluminescence (PL) spectra of **(B)** CQDs-N10-Y, **(C)** CQDs-N20-G, and **(D)** CQDs-N30-B at different excitation wavelengths.

The optical properties of as-prepared CQDs were explored by UV-vis absorption and photoluminescence (PL) spectroscopy. The UV-vis absorption spectra of as-prepared CQDs are shown in Figure 6A. It is clear that there are a strong peak and a weak peak at 221 and 298 nm corresponding to π-π^*^ transition of C=C and n-π^*^ transition of C=O, respectively (Dehghani et al., 2018; Yang et al., 2018). Apparently, the shoulder peak at 298 nm is almost invisible in the spectrum of CQDs-N10-Y, indicating that the surface content of COOH is relatively lower in CQDs-N10-Y (Zhang et al., 2016). This phenomenon is consistent with the results of XPS analysis. The adsorption of CQDs-N30-G occurs mainly in the UV region (<400 nm). However, the absorption regions of YCQDs-N10-Y and CQDs-N20-G extend to the visible region (400–650 nm), and the adsorption is more prominent for CQDs-N10-Y, suggesting more narrowing of the bandgap (Choi et al., 2018).

The photoluminescence (PL) spectra of the as-prepared CQDs excited at the different wavelengths are shown in Figures 6B–D. The maximum emission peak shifts from ~525 to ~450 nm as the concentration of H_2_O_2_ increases from 10 to 30%. Moreover, it is worth noting that like most CQDs (Zhu et al., 2013; Liu Q. et al., 2018; Chen et al., 2020), the PL behavior of CQDs-N30-B exhibits the emission wavelength is dependence of the excitation wavelength and the intensity of PL decreases with increase of excitation wavelength. But the emission peaks lie in almost the same wavelength (~510 or ~525 nm) with increasing excitation wavelength for both CQDs-N10-Y and CQDs-N20-G. Two distinct emission modes were proposed to interpretive the PL mechanism of CQDs, i.e., intrinsic emission mode and extrinsic emission mode (Liu et al., 2013). The emission peak near 450 nm when excited near 325 nm is considered as the intrinsic emission of sp^2^ carbon hexagons. The emission peak near 500 nm when excited with 325 nm and above is attribute to the extrinsic emission due to the defects of CQDs including oxygen-containing functional groups or sp^3^ carbon. The work of Yoon et al. (2016) indicated that oxygen-containing functional groups may create new energy states (extrinsic state) inside the band gap of CQDs resulting in the red shift of PL emission. The results of FTIR and XPS analysis show quantities of oxygen-containing functional groups exist on the surfaces of CQDs-N10-Y, CQDs-N20-G and CQDs-N10-B, but the amount of sp^3^ carbon is very low. Therefore, the emission peaks near 520 nm of CQDs-N10-Y and CQDs-N20-G can be attributed to extrinsic emission mode due to the new energy states created by oxygen-containing functional groups. But it is interesting to note that although the oxygen content of CQDs-N30-B is the highest in all the as-prepared CQDs, the emission peak of which is still near 450 nm excited near 325 nm. We propose that this is because in the case of smaller size of CQDs-N30-B (3.2 ± 0.9 nm) the emission is likely dominated by the intrinsic transitions rather than the extrinsic, oxygen defects related transitions. So, our results suggest that for small CQDs prepared by the method described here both intrinsic and extrinsic transitions contribute to the observed CQDs PL, where the former dominate the PL in CQDs-N30-B and the latter dominate the PL in CQDs-N10-Y and CQDs-N20-G.

## Conclusions

In conclusion, we have developed a facile, fast, and green method to prepare bandgap tunable CQDs solely from anthracite. The emission color of CQDs can be adjusted from yellow to blue under UV light. The bandgap change of as-prepared CQDs can be achieved by simply controlling the concentration of H_2_O_2_. The morphology, size and PL properties of the as-prepared CQDs indicate that the blue luminescence may originate from the intrinsic emission, but the yellow and green luminescence may originate from the extrinsic emission due to the new energy states created by the oxygen-containing functional groups inside the band gap of CQDs. This novel strategy for fabricating optically tunable CQDs from coal is highly promising for the high-end application of coal.

## Data Availability Statement

The datasets generated for this study are available on request to the corresponding author.

## Author Contributions

QL, CZ, and JJ conceived and designed the experiments. YS, JC, YZ, and GH fabricated and characterized the sample. BX, LZ, GH, and YC analyzed the data. All authors discussed the experiment results and contributed to the writing of the paper.

### Conflict of Interest

The authors declare that the research was conducted in the absence of any commercial or financial relationships that could be construed as a potential conflict of interest.
